# Supplementary figures and images for: SP1 and NFY Regulate the Expression of PNPT1, a Gene Encoding a Mitochondrial Protein Involved in Cancer
Source: Int J Mol Sci. 2022 Sep 27;23(19):11399. doi: 10.3390/ijms231911399 (PMC9570217; doi:10.3390/ijms231911399)

chr2:55693349-55694242

Fold change over control

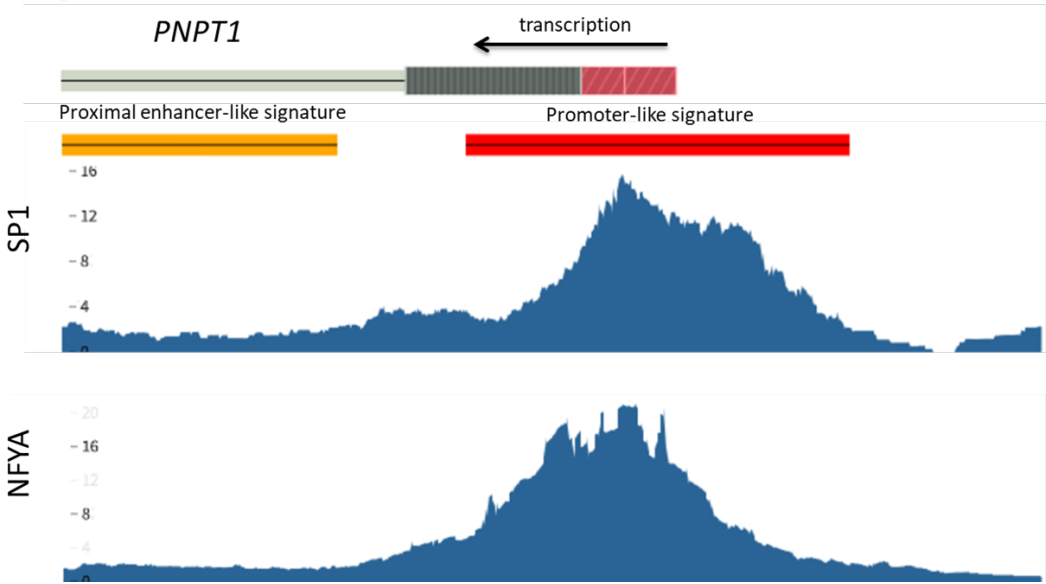

Supplement: Supplementary file 1 [file ijms-23-11399-s001.zip › Figure S1.pdf]

Figure S2: Summary figure

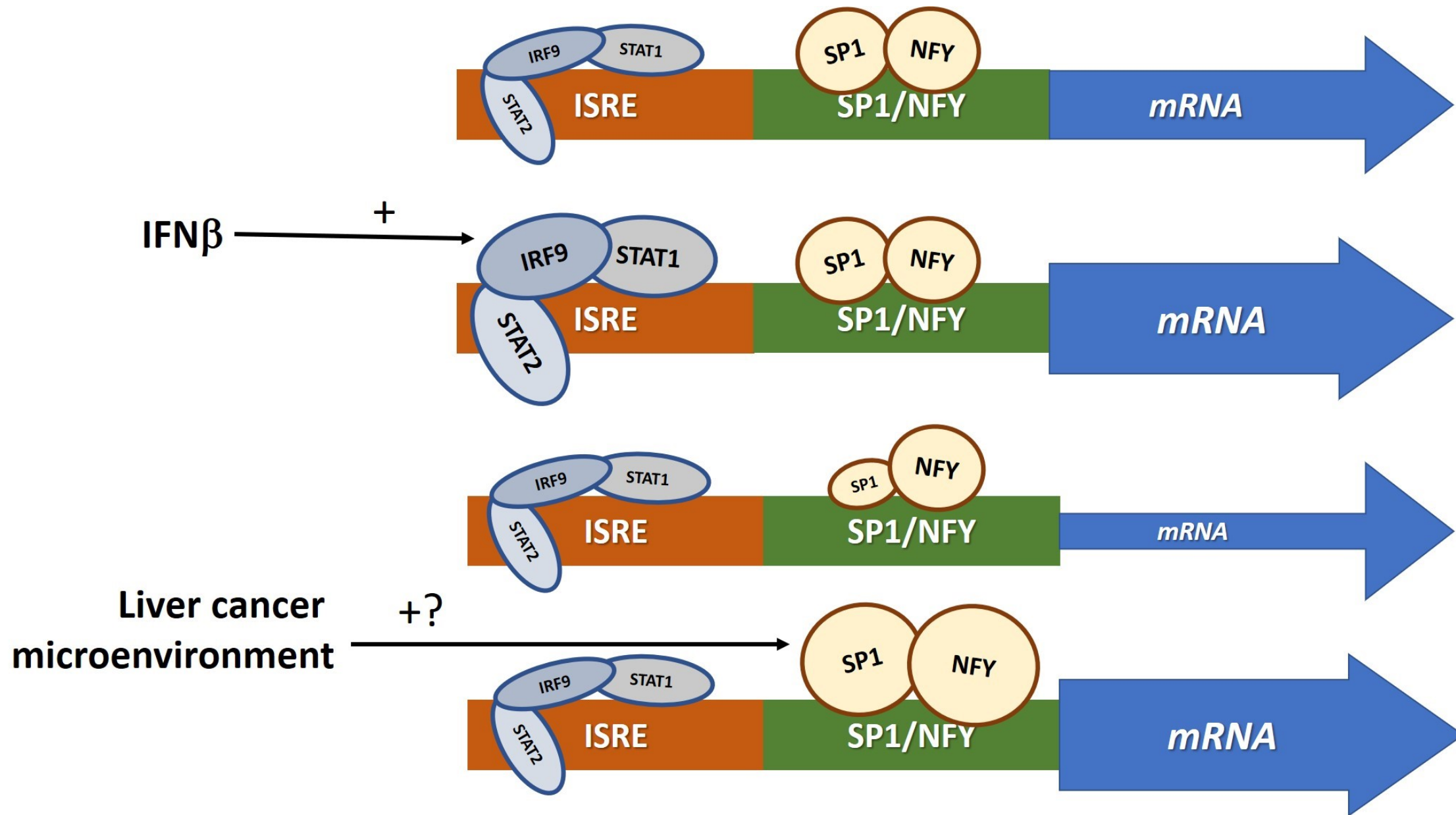

Supplement: Supplementary file 1 [file ijms-23-11399-s001.zip › Figure S2.pdf]
